# Where am I? Who am I? The Relation Between Spatial Cognition, Social Cognition and Individual Differences in the Built Environment

**DOI:** 10.3389/fpsyg.2016.00064

**Published:** 2016-02-11

**Authors:** Michael J. Proulx, Orlin S. Todorov, Amanda Taylor Aiken, Alexandra A. de Sousa

**Affiliations:** ^1^Crossmodal Cognition Laboratory, Department of Psychology, University of BathBath, UK; ^2^European Network for Brain Evolution ResearchThe Hague, Netherlands; ^3^Department of Philosophy, University of DurhamDurham, UK; ^4^School of Society, Enterprise and Environment, Bath Spa UniversityBath, UK

**Keywords:** spatial cognition, social cognition, navigation, personality, reference frames, allocentric frame of reference, egocentric frame of reference, cognitive neuroscience

## Abstract

Knowing who we are, and where we are, are two fundamental aspects of our physical and mental experience. Although the domains of spatial and social cognition are often studied independently, a few recent areas of scholarship have explored the interactions of place and self. This fits in with increasing evidence for embodied theories of cognition, where mental processes are grounded in action and perception. Who we are might be integrated with where we are, and impact how we move through space. Individuals vary in personality, navigational strategies, and numerous cognitive and social competencies. Here we review the relation between social and spatial spheres of existence in the realms of philosophical considerations, neural and psychological representations, and evolutionary context, and how we might use the built environment to suit who we are, or how it creates who we are. In particular we investigate how two spatial reference frames, egocentric and allocentric, might transcend into the social realm. We then speculate on how environments may interact with spatial cognition. Finally, we suggest how a framework encompassing spatial and social cognition might be taken in consideration by architects and urban planners.

## Introduction

Understanding the nature of spatial knowledge is a classical issue in psychology, philosophy, and behavioral biology; it is also a core practical problem for architects designing effective buildings. Heidegger (1962) suggested that human beings are always *spatial* beings when addressing how we find ourselves in the world, a space shaped by the activities we engage in. Such a view might appeal to adherents of embodied cognition, who take the perspective that thought itself is emergent from the interaction between the environment, the body, and the mind (Wilson and Golonka, 2013). Our interaction with space may also define what human beings are capable of, and how we define ourselves. In modern times investigations on the attributes of spatial cognition receive a considerable amount of attention (Klatzky, 1998; Burgess, 2006; Mou et al., 2007), and best practice in architecture and urban planning requires knowledge of spatial cognitive abilities to create practical, safe, efficient, and accessible environments (Marquardt and Schmieg, 2009; Marquardt, 2011).

Knowing the location of the objects around and how to reach them is crucial for carrying out the vast majority of organism's activities. The nature of an organism's plastic spatial relationship to the environment is also a defining quality of its identity. As mobile beings, we rarely remain in one place for a long period of time. It is imperative to our physical and social survival that we travel to different locations to perform daily tasks, visit with family and friends, and exercise. These tasks require an accurate spatial representation of our environment for proper navigation, perhaps through a *cognitive map* (Tolman, 1948). Real-world navigation requires a combination of cognitive skills such as object recognition (what), localization (where), and obstacle avoidance (how; Maguire et al., 1999). Theories of embodied cognition do not include a role for representations, though grounded cognition (Barsalou, 2008) allows for representations which are grounded in perceptual experience.

The perception of space has long been seen as a special task for perception in general, and vision in particular. Already a century ago Helmholtz (von Helmholtz, 1962), and later Gibson (Gibson, 1950), were intrigued, physiologically and psychologically, by the origin of spatial cognition and the role of vision. David Marr wrote in his book *Vision* that the common definition (and Aristotle's, too) for seeing is to “know what is where, by looking” (Marr, 1982, p. 3). Sight is the primary sense that humans use to know not only what is where, but also to know the locations of things in relation to the self and others. One major question in perceptual research is how our visual system is able to create this spatial representation and subsequently perceive distances between ourselves and other objects. Thus, much of what we understand about human spatial perception has taken a visual sciences approach. However, vision is only one way of experiencing space; research has suggested that other senses represent space differently. What are the implications of individual differences in spatial perception?

**Figure 1:**
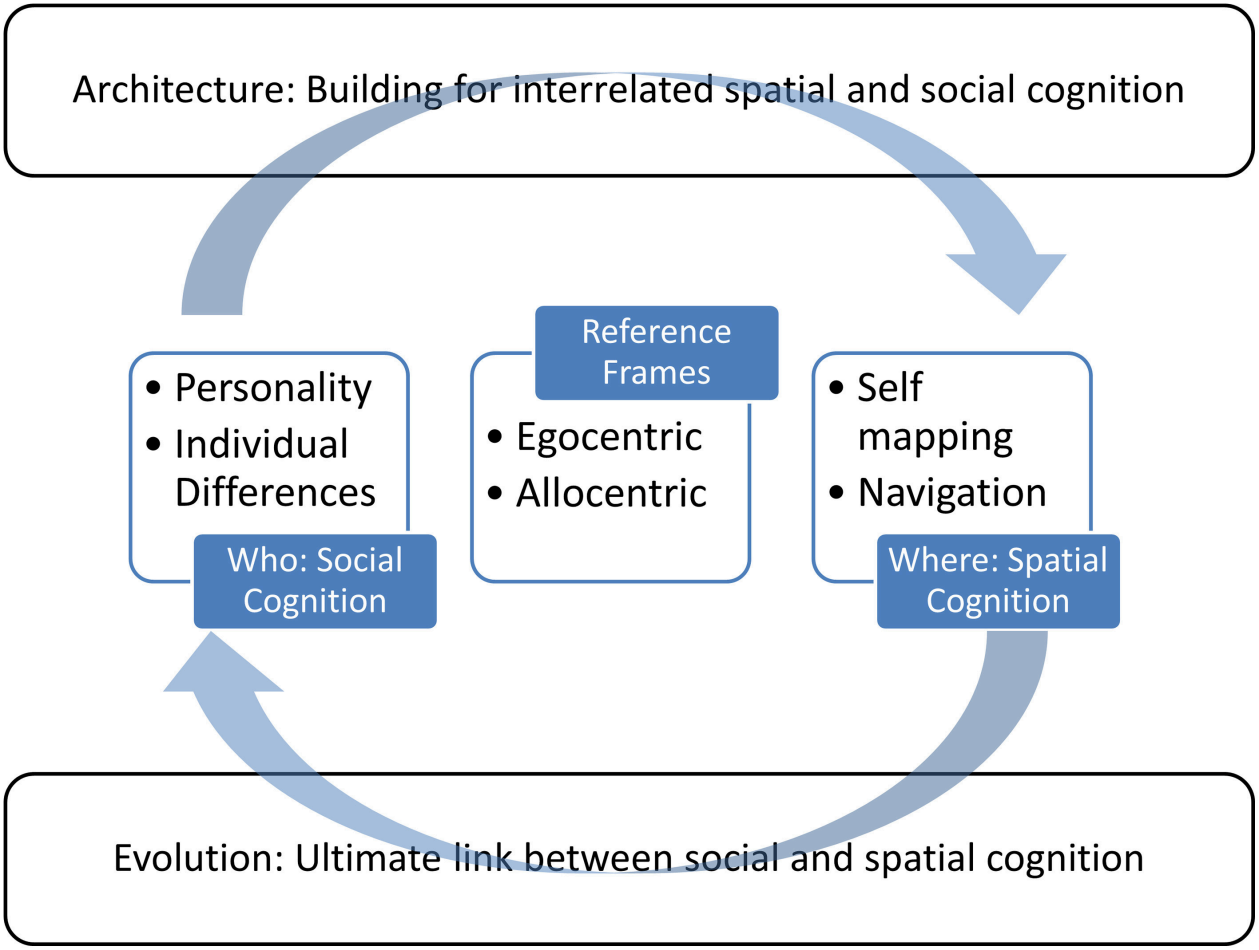
**An illustration of the approach taken in this review**. Central to the review are the concepts of self and spatial cognition, which are connected by the consideration of egocentric and allocentric reference frames. These provide the context to move from a foundation in evolutionary theory to applications for the built environment.

With a grounding in evolutionary theory, we review the role of individual differences in spatial tasks, and conversely, how the built environment can restrict or promote spatial cognition, and how that influences one's selfhood. Figure 1 provides a schematic of our approach in this paper. We aim to influence the building of environments to take into account individual differences, ability, and disability. Critically, traditional discipline-based approaches are insufficient to create a breakthrough in revealing the relationship between spatial cognition and the self, to detail the full implications for self-identity and application for the built environment, and to start by clarifying the terms as used in each field. First, we provide clear cross-disciplinary definitions of terms linking these concepts. “Egocentric” and “allocentric” are terms that are used quite broadly in academic literature, but it is not clear that their usage is consistent across disciplines. In some cases, different usages may be due to simply using the same label for a different construct. For example, the term “egocentric” in particular is most often used to describe a personality type rather than a spatial reference frame. The term “allocentric” generally has only a spatial use, in terms of defining object locations to one another, rather than in relation to the self in an egocentric reference frame. The dual use of these terms in spatial and personal contexts may have arisen by chance. However, theories of grounded, and even embodied, cognition suggest that the similarity of the definitions could actually imply a common ontology, which in turn could reveal shared concepts and mechanisms (Barsalou, 2008). That is discrepancies in the usage of these terms might actually reveal a shared underlying connection with a latent relation between spatial processing and aspects of individual differences and self-awareness. Therefore, this paper explores whether who we are is defined by where we are. “Who” is considered in the concepts of the ego, the self, the social self, and in personality. “Where” is considered on various spatial scales and environments.

We explore whether seemingly different intended usages of the terms egocentric and allocentric are in fact related, in terms of neuroanatomical or behavioral attributes. Thus, a major goal of this paper is to review how these terms have been used, and clarify what these terms actually mean in theoretical, experimental, evolutionary, and applied contexts (cf. Proulx et al., 2014). There are a myriad of ways in which our spatial coordinates and our “selves” are intertwined. Investigating the self in humans and non-human animals from social and spatial perspectives may help explain how spatial factors interact with personality, self, and perception. This framework has an important broader impact. Optimizing spaces for the wellbeing of all is a critical job for architects and urban planners. This paper, through the interacting topics illustrated in Figure 1, demonstrates how architecture might take guidance from data about how the mind works from fields such as neuroscience, psychology, philosophy, anthropology, and behavioral biology.

## Ego: Defining the self and its place in space

### How the ego creates the self: A product of personality and the environment

Why do individuals respond to environmental stimuli differently? How do our interactions in social and spatial environments relate to who we are? In this section we first provide an overview of personality theories, and then expand this into an overview of theories about ego, self, and personality. Thus, we describe a conceptualization in which the self is determined by an interaction between an individual's personality and environmental factors (including social and spatial factors). We further develop this to consider how extreme differences in environment and sensory ability could play a role in shaping the self. Finally, we introduce the terms egocentric and allocentric from the perspective of personality theory.

The roles of the ego, self, and personality as constituents of the mind have been a hot topic of debate over the last century, with definitions and postulations regarding their unity, diversity and function, changing with time and with shifts in paradigms (Baumeister, 1987). Currently, a contemporary convergent view has emerged linking self, personality, and the mind. The *mind* has been defined by Minsky as “what brains do” (Minsky, 1988), that is, thinking, understanding, and learning (Perner, 1991), and the *self* as a product of the mind, proposed to emerge as phenomenon related to the experienced, sensed, and understood (Demetriou and Kazi, 2001). According to that same view, *personality* provides the dispositions through which an organism perceives and interacts with the world, and as such provides a framework for the mind to operate within (Ferrari and Sternberg, 1998). Therefore, the self can be thought of as a reference frame of the mind derived from the interaction between personality dispositions and the environment. The ego is that which creates the self, and these are a product of personality and the environment (Lewin et al., 1936; Wayment et al., 2015).

In their recent work on “the quiet ego” self-identity, Bauer and Wayment find that in modern scientific psychology the term ego is either synonymous with the term self, or it describes the process which creates the self (Bauer and Wayment, 2008). They prefer defining the ego as a process, and we also find that this leads directly to the approach that the ego is a frame of reference in both social and spatial interactions. Wayment et al. elaborate on the conceptualization of the ego as a frame of reference for the self by pointing to parallels between the terms “ego” vs. “self,” and William James' concepts of “I” vs. “Me”: just as the I frames the Me, the ego frames the self (Wayment et al., 2015). According to their working definition, the *ego* is that which does the thinking, and the *self* is produced by the ego (including one's conceptions of physical self, social self, and psychological self). It is this extension of James' definitions which we feel best applies in the current context.

There are multiple (but not necessarily exclusive) ways of understanding the self which are relevant to personality, and to social and spatial cognition. Here we review the classic theories of the self in an historical perspective as these serve as the foundation of modern studies of the self, and provide the clearest links to philosophy and behavioral biology. Important steps toward understanding and defining the self are, first, clearly defining personality and, second, demarcating the domains of operation of the mind. The latter is comprehensively covered by the concept of cognitive abilities: the adaptive abilities related to knowledge acquisition, understanding, and learning (Jensen, 1998). Defining personality is perhaps an even more daunting task, which can incorporate different fields of study (for example, the aim could be either scientific or spiritual; perspectives can take from zoology or be strictly human-centered), and can vary also according to the desired scope and rigidity of the definition.

Given that the initial interest in the study of personality was inspired by research in humans, in one of the earliest attempts, Allport defined it as “the dynamic organization within the individual of those psychophysical systems that determine his characteristics, behavior, and thought” (Allport, 1937). He suggested that personality might be biologically determined at birth, but also shaped by environment and experiences. Another well-known, early attempt to describe personality's development and structure came from Freud (1923). He recognized the role of development on personality and proposed several sensitive developmental stages modulating the set of personality traits. One of his contributions was the delineation between the *id*, the *ego*, and the *super-ego*, as the main constituents of human personality—*id* (Latin: “that”) was defined as inherited biological (unconscious) tendencies and drives of the personality, the *super-ego* was defined as incorporating the morals and values of the society which one acquires throughout development from their social sphere, and the *ego* (Latin: “I”) was defined as the result of the interplay of these two processes—mediating and modulating id's desires according to the requirements of the super-ego (Freud, 1923). Thus, Freud acknowledged the fact that personality has both public (super-ego) and personal (ego) aspects that one might be consciously aware of, and some of which are inherently unknowable to the individual (those covered by the id).

Another attempt to define and deconstruct personality was trait theory. One of its proponents, Eysenck, proposed that personality might be explained by as few as three general, orthogonal, personality traits—namely extraversion, neuroticism, and psychoticism (Eysenck, 1963). Eysenck attributed personality differences to differences in the functioning of the autonomic nervous system dependent on the balance and specifics of neuronal excitation and inhibition, and thus moving closer to a neurobiological framework for understanding personality differences (Hegerl et al., 1995). He recognized personality differences as dispositions influencing how one relates and interacts with the world and his theory was developed into the Big-Five personality model (Digman, 1990), comprising of the following five traits: Openness, Conscientiousness, Extraversion, Agreeableness, and Neuroticism. Thus, far, the Big-Five model is accepted as the most comprehensive and concise model, explaining most of the variation of human personality traits (Digman, 1990).

After acknowledging that personality differences might be ubiquitous in animal life and not exclusively human property, Gosling (Gosling, 2008) defines personality as “those characteristics of individuals that describe and account for temporally stable patterns of affect, cognition, and behavior” (Gosling and John, 1999). Among researchers working on non-human animals, the term behavioral syndromes is used preferentially, but yet, interchangeably with the term personality in human studies (Gosling, 2008). Following the steps of personality research in humans, the trait approach has become dominant in the studies in non-human animal, too. Such studies have so far identified several stable individual traits, that are also expressed across different populations in numerous species, such as the shy–bold axis (Sloan Wilson et al., 1994; Coleman and Wilson, 1998), the proactive–reactive axis (Koolhaas et al., 1999), and exploratory behavior (Verbeek et al., 1994). Thus, personality differences have been used to describe the predictable behaviors of individual non-humans as well, in species ranging from orangutans (Weiss et al., 2011) to honeybees (Liang et al., 2012).

The concept of the self is much more recent than the concept of personality, both in terms of it being a recent philosophical consideration, and it terms of how it arises from the natural world. It has been suggested to be a “created problem”—one arising from the pursuit to define and redefine identity over time (Baumeister, 1987) and thus difficult to define unequivocally. Whereas, personality can be directly grounded in natural phenomena, the self seems to be an emergent phenomenon: a product of the interaction of the evolved capability of self-awareness and other environment-oriented, domain-specific representational, and interaction systems, involved in conceptualizing relationships in the environment in both the cognitive and the social domains (Demetriou, 2003). Because self exists at that intimate level, the modifier “self” is often used as an adjective to describe the individualized experiences of the person. It is related to concepts like self-awareness, self-esteem, self-knowledge, and self-perception and was defined by Baumeister (1987) as emerging “at the interface between the inner biological processes of the human body and the sociocultural network to which the person belongs.”

A turning point in conceptualizing the self was the theory of Maslow (1968), in which he emphasized the importance of self-fulfillment. Derived from the writings of Heidegger (1927) Maslow proposed that the present self is defined by its dynamism; it is defined in terms of the efforts made toward changing into its future form. He suggested that the self has potentialities for future existences, and only by understanding these so called self-actualization drives, one can truly understand the self. Thus, the concept of potential fulfillments is thought to give meaning to the present self, and in that way the self can be thought of as a “plan” or a motive for fulfillment of potentials, based on the state of the current self and on personality. A number of recent reviews have discussed recent advancements of theories of the self (Gilovich, 2002; Forgas and Williams, 2014), though many of these take root in the same historical review we have provided here.

Both spatial and social behaviors directly impact the self. Firstly, in the aforementioned modern view, the self is considered to be a product of the representational and interaction systems of the organism and its environment (including spatial and social factors), as modulated and mediated by the personality. Further, in self-aware individuals, the self is a product of the ability of the organism to be aware of mental states and attention in others (Reddy, 2003). Demetriou (2003) proposed that the self is the product of several factors which he classifies as environment-oriented systems which are comprised of six different functional modules of cognitive functioning—categorical, quantitative, causal, spatial, propositional, social, and pictographic system.

The self, understood as a reference frame of the mind derived from the interaction between personality dispositions and these environment-oriented systems, is thus a product of the individual's social and spatial interactions, among others (Humphrey, 2011). Just like one's categorical or propositional systems influence one's ideology and the way one sees and processes its environment (Lakoff and Johnson, 1980; Tversky and Kahneman, 1981), spatial and social systems, too, have a crucial role in understanding the self, and its potentials which it is striving to fulfill. Thus, a crucial step toward understanding the essence of the self is to delineate and elucidate the specific social and spatial strategies, and their interaction with personality. Such consideration can provide a means for the individual to fulfill the potential of their self in the most optimal way.

By extension of this framework, extreme differences in environment or sensory ability could, along with personality, have a significant interaction with the self. First, sensory disabilities (such as blindness and deafness) and environmental constraints (such as solitude or confinement) can modify an individual's interaction with the environment, in particular by preventing social and spatial interactions and limiting perception. In addition, they could have an impact on personality, and thus have an impact on the self in this manner. Second, personality influences how the external environment is perceived. Much research and theoretical development has noted the role of attention for consciousness (Dehaene et al., 2006) [though admittedly controversial Van Boxtel et al., 2010], such that one is consciously aware of only that to which one pays attention, whether with simple visual search task (Joseph et al., 1997), or more complex video displays (Simons and Chabris, 1999). Personality traits influence what one attends to. For example, in emotion-induced blindness, there is impairment in visual processing due to attention to an emotional stimulus. The degree to which emotional stimuli can alter visual attention is directly related to a personality trait: harm avoidance (Most et al., 2005). Thus, one's personality creates a reference frame, via attentional prioritization, of what is valued, and therefore attended to, in the environment (Anderson et al., 2011).

The ways in which we sense and perceive the world could influence the sense of who we are in personality and social domains. Although there have not been reports of different personality dimensions for visually impaired persons, higher levels of anxiety have been noted (Donoyama and Takeda, 2007; Donoyama and Munakata, 2009), and this has been shown to influence the expression and character of one's personality. Thus, although visual impairment does not influence personality *per se*, it can affect the expression and perception of personality linked behaviors (Zahran, 1965; Coren and Harland, 1995; Reid, 2000), and provides an important method for examining whether visual experience rather than innate mechanisms shape social and spatial behavior. Further, sensory abilities influence social interactions—standard social interaction is multisensory, though often driven by visual experiences such as facial, emotional, and body expressions. Although, as discussed, personality is influenced by several systems, the social system is largely the focus, because personality is visible in the social domain. For example, according to Furnham, personality comprises the correlated states, traits, and expectations of behavior in social settings (Furnham, 1992).

### Egocentric or allocentric: Individual differences in spatial reference frames and personalities

The terms “egocentric” and “allocentric” are used in both social and spatial domains. Theories of grounded cognition are built on the notion that mental concepts and representations are not merely semantic, and thus amodal, but instead are grounded in sensorimotor experience and interactions with the environment (Barsalou et al., 2003; Barsalou, 2008). Such grounding implies that there could be a common source for these reference frames in both the social and spatial domains. Here we define reference frames in the spatial sense, review evidence for individual differences in reference frames in cognition, and then describe how reference frames interact with self-perception and personality.

**Figure 2:**
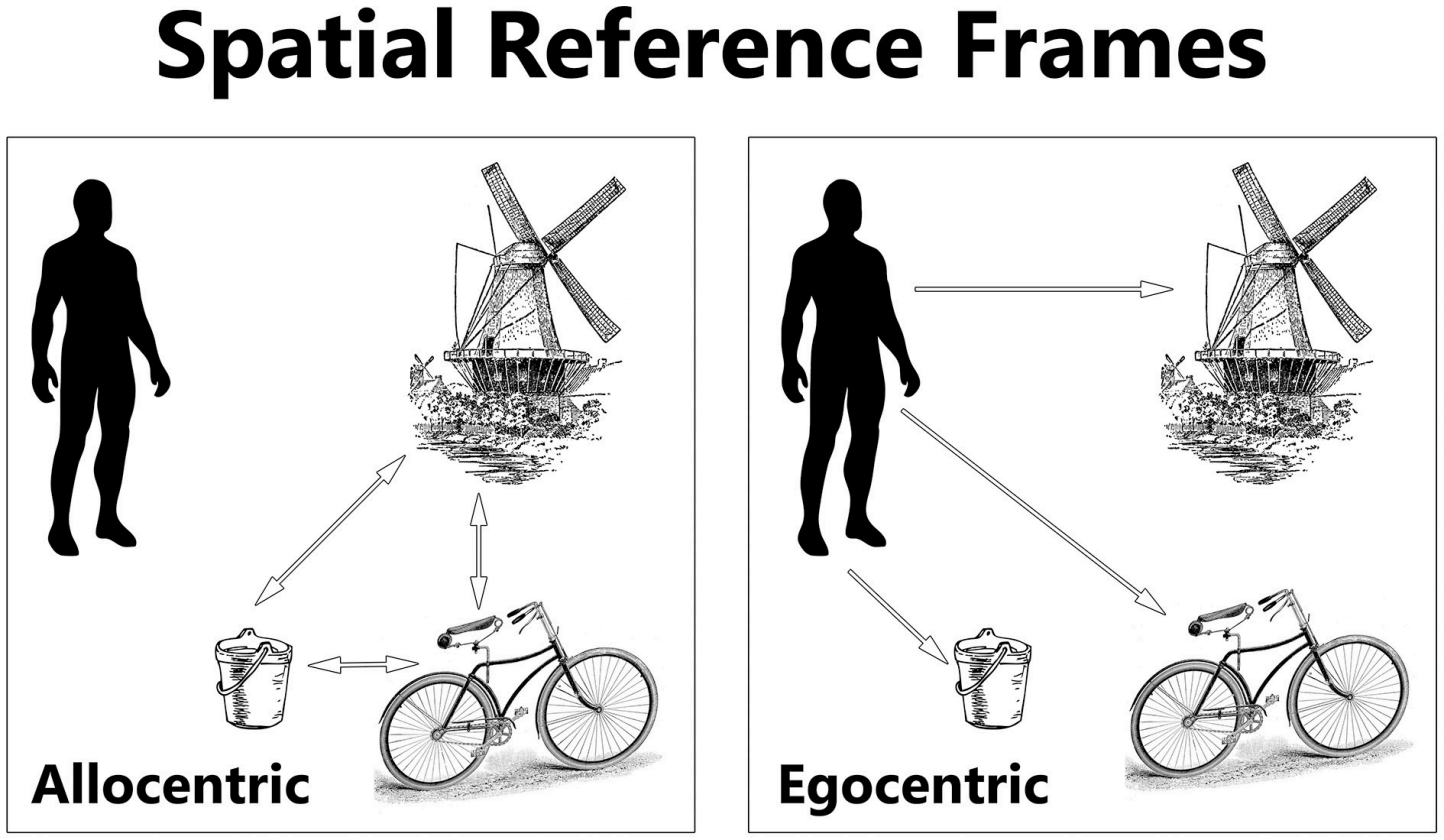
**The location of objects can be represented in egocentric and allocentric spatial reference frames**. An egocentric reference frame represents objects in relation to the location of the self (the observer). An allocentric reference frame represents objects in relation to one another. Inspired by Kozhevnikov (2010).

The concept of a *frame of reference* has is origins in describing spatial coordinates and is still used in physics to describe “a system of coordinate axes in relation to which size, position, or motion can be defined” (Dictionary, 2015). However, this term has since been broadened in its usage to describe an aspect of a cognizant being, whose frame of reference (or reference frames, frames of mind, and framing) influences behavior. The ways in which one can mentally represent the locations of things are called *spatial reference frames*. Egocentric and allocentric are two general forms of spatial reference frames (see Figure 2). An egocentric reference frame is where one denotes the location of something else in reference to oneself. In contrast, an allocentric reference frame is where the location of something else is in reference to yet another object, independent of oneself (Byrne et al., 2007). For example, a person in the UK could define the location of France compared to himself in an egocentric sense, or instead define the location of France compared to Germany instead, in an allocentric sense. The reference frame used may interact with other, non-spatial domains of cognition, such as social cognition.

There has been much discussion about how, in practice, space is actually represented cognitively in humans and other animals, and whether one of the frames of reference dominates, or they necessarily work together (Wang and Spelke, 2002) (Burgess, 2006). These reference frames interact to guide behavior, but can also be preferentially used depending on the conditions of one's environment. Different circumstances seem to favor egocentric and allocentric approaches differently (discussed in Section Environmental Influences on Spatial Reference Frames; Byrne et al., 2007). Byrne and colleagues describe in their model how spatial cognition involves separate egocentric and allocentric neural pathways working together (discussed in Section Social and Spatial Cognitive Neuroscience). Briefly, egocentric reference frames appear to be supported by a number of cortical areas, from primary sensory processing such as retinotopic maps from primary visual cortex through to posterior parietal cortex (PPC), including the precuneus and retroplenial cortex (Byrne et al., 2007). In contrast, allocentric reference frames require the representation of environmental locations with support by the hippocampus and parahippocampal regions, with interactive connections to the egocentric representations available through retroplenial cortex (Byrne et al., 2007). In addition, species-level differences are possible, and might be based on different ecological pressures. Finally, there is increasing evidence for individual differences in terms of egocentric and allocentric strategies used to relate to the embodiment of the self in space and during spatial navigation.

Sensory abilities influence the use of spatial reference frames. The default spatial reference frame can differ among individuals as a function of visual experience (Pasqualotto and Proulx, 2012; Pasqualotto et al., 2013). Although those with visual experience tend to use an allocentric (object-based) reference frame, those without visual experience use an egocentric (self-based) reference frame to remember object locations. For example blind people preferentially navigate space using an egocentric, route-based perspective (Pasqualotto and Proulx, 2012; Pasqualotto et al., 2013). This is consistent with the preference for an egocentric route-based approach to navigation by blind persons who are less efficient in using allocentric map-based approaches or representations (Steyvers and Kooijman, 2009).

How does sensory information inform spatial reference frames? Different sensory modalities provide different types of information about space. Vision is the sensory modality most relied upon for distal information. This may be demonstrated in how visual perception influences the perception of distance. With a consistent, unaltered ground, sighted individuals are just as accurate at walking to previously viewed objects when their eyes are closed compared to when they are open, even at large distances of up to 22 m (Sinai et al., 1998). However, various visual characteristics such as surrounding environment (Lappin et al., 2006), ground surface structure (Sinai et al., 1998; He et al., 2004), and ground slant (Stefanucci et al., 2005) can bias these judgments. These different measures are useful for determining the extent of embodiment and the mechanisms as well (Wilson and Golonka, 2013). Further, even in cases where visual stimuli can be perceived, it may be possible that a later deficiency in multisensory integration or processing could interfere with spatial perception. An example of how spatial perception is embodied as self-perception is the case of an eating disorder. The “Allocentric Lock Hypothesis” suggests that perhaps a person with anorexia nervosa views one's own body from an allocentric spatial perspective by default, and neural-rewiring may pose a restriction on the egocentric spatial perspective (Riva, 2012; Riva and Gaudio, 2012). Egocentric perception of one's body should necessarily be multisensory in nature, with visual, somatosensory, kinesthetic, and interoceptive cues providing an integrated percept; in contrast the allocentric perception of oneself is largely driving by visual perception via reflections or mental representations independent of self-perception and therefore would not correct distortions in interpreting body perception with other sensory cues. Thus, the spatial reference frame could directly influence the perception of self.

Egocentric and allocentric are often described as aspects of personality or social behavior. *Egocentrism* uses *ego*, the Latin word for I; in egocentrism that which thinks is the focus of what it thinks about, and the frame it thinks through. The modern conceptualization of egocentrism is attributed to Piaget, who at first drew upon the influence of Freud while doing scholarly work on psychoanalysis (Kesselring and Müller, 2011). Piaget described egocentrism in terms of developmental psychology, as an aspect of an early stage in cognitive development (Piaget, 1936). According to Piaget, young children are egocentric: they ascribe their own feelings and experiences to everyone. This usage of the term has since undergone some modifications, by Piaget himself, and more broadly in psychology. As Piaget himself emphasized, the concept of egocentrism is different from egocentrism in ordinary language, but rather, “Cognitive egocentrism, as I have tried to make clear, stems from non-differentiation between one's own and other possible points of view and in no way at all from an individualism which precedes relations with other people” (Piaget, 1995).

Egocentric social behavior is generally considered to be relatively simple and an early stage of behavioral progression, not just in terms of human development, but also in terms of behavioral evolution, since it seems to be phylogenetically primitive to empathetic behavior. According to the model of Koski and Sterck, chimpanzees demonstrate aspects of empathy including emotional mimicry, a distinction between self and other, and the understanding of mental states of another without facial cues (although as is the case in humans there exist individual differences in these capacities within this species Koski and Sterck, 2009). However, chimpanzees are more egocentric compared to humans because chimpanzees do not have the human capacity for cognitive empathy, which involves such abilities as understanding past, conflicting and hidden emotions in other individuals (Koski and Sterck, 2009). Indeed, empathy deficits, such as those arising from narcissism, can directly influence one's perception of the world (Jankowiak-Siuda and Zajkowski, 2013). Consistent with the discussion in a prior section on the link between spatial and social reference frames, the key neural structures for empathy such as the insula and anterior cingulate cortex are also implicated in salience, or the assignment of attentional priority to sensory information (Frith and Frith, 2003; Decety and Jackson, 2004; Seeley et al., 2007). The relative focus of an individual on oneself or on others might relate directly to basic sensory information processing (Critchley et al., 2004; Avenanti et al., 2006) in the same way one might focus on spatial information.

The term *allocentrism* (from Greek *allos*, other) has its origin in visuospatial cognition. However, allocentric has also been used in describing social behavior, where allocentrism is a “tendency” related to the Big Five personality traits (Triandis et al., 1985). Interestingly, in social behavior the term allocentric is contrasted with the term *idiocentric* (rather than egocentric), apparently borrowing from Freud's use of the term *id* (Triandis et al., 1985). Allocentrism and idiocentrism are considered to be psychological manifestations of personality at the cultural level, corresponding to cooperation vs. individualism, respectively (Triandis et al., 1985). In contrast to the opposition of egocentricism and empathy described above, allocentricism is a necessary component of empathy. Being able to take the perspective of another is essential to express empathy, though it is not sufficient because one could understand another's perspective without experiencing the emotional feelings that the other is experiencing (Eisenberg, 2000).

As mentioned above, the terms egocentric and allocentric are both used to describe social behavior independently, but are not so often used as a pair. However, Frith et al. (Frith and de Vignemont, 2005) draw upon the definitions of egocentrism and allocentrism as used in describing spatial behavior to similarly classify social behaviors. They emphasize the difference between the ways in which these terms are used in spatial vs. social studies. They use this to show that, in spatial behavior, egocentrism, and allocentrism are complementary: you can switch between egocentric and allocentric maps in a wayfinding task. In social behavior they also tend to be complementary. Those with ASD however lack the task switching ability between allocentric and egocentric social perspectives—an idea which challenges the more simplistic notion that ASD is simply a severe case egocentrism (Frith and de Vignemont, 2005).

Training might also impact whether one attends more to one's self, or via different reference frame, to other aspects of the environment. In meditation one acts to control one's own cognitive faculties, in particular attention and emotion (Lutz et al., 2008). Thus, one's frame of reference might be altered within an individual independently of ability, personality, or spatial and environmental pre-conditions. The techniques set out to cause changes in frame of reference, mostly related to social behavior and the sense of self, and perhaps these could also be related to changes in spatial frames of reference. Meditators perform more effectively on visual memory and spatial skills tasks immediately after deity yoga meditation, which focus on mental imagery (Kozhevnikov et al., 2009), and have more gray matter in the subiculum of the hippocampus (Luders et al., 2013), a substructure with functions related to spatial cognition and stress modulation (O'Mara, 2005). Mindfulness practice involves attentional training for the ability to be aware of one's own thoughts, feelings, and experiences in general and non-judging terms (Kabat-Zinn, 1990) and has been related to faster reaction time on a mental rotation task (Geng et al., 2011). As spiritual and clinical practices, they strive for an impact on behavior. Ultimately, such practices strive for non-egocentric, allocentric-like effects on personality such as a “quiet ego” self-identity (Wayment et al., 2015). An “allo-inclusive” identity which incorporates the social and physical environment into the perceived sense of self is a desired identity because such individuals have lower depression and higher satisfaction, and feel more connected to others (Leary et al., 2008).

## The overlap in social and spatial behavior, and its neurophysiological basis

### Allocentric and egocentric aspects of social and spatial distance

Despite the overlap in the definitions for egocentrism within the social and spatial spheres, traditionally the fields of spatial and social cognition are independent. Nonetheless, a few recent studies have discovered that the spatial and social domains interact. Recent findings that spatial strategies vary according to sociability fit well with increasing evidence for embodied theories of cognition, where mental processes are grounded in action and perception (Barsalou et al., 2003). Individuals vary in personality, navigational strategies, and numerous cognitive and social competencies. Who we are might be integrated with where we are, and impact how we move through space. Studies of navigation have been particularly fruitful for understanding the role of egocentric and allocentric reference frames in spatial navigation (Passini and Proulx, 1988; Passini, 1996; Pasqualotto and Proulx, 2012; Pasqualotto et al., 2013).

Several studies imply that individual differences in representation are possible and might arise from other forms of experience. Although social behavior does not immediately seem spatial in nature, some studies have linked social skills and spatial skills. Work by Shelton et al. (Marchette et al., 2011; Shelton et al., 2012) found that social skills predicted accuracy in a spatial perspective-taking task when the task included a potential agent rather than an object. This finding implies that our sense of location depends on an interaction between both the social self and the geometric representation of our body in space in relation to other people or things.

Spatial reference frames are the fundamental way that the locations of objects and oneself are represented in an environment. Similar frames of reference are used in social contexts as well, such as by using a spatial metaphor such as a vertical hierarchy to define place and role within a group (Rodman, 1999). Many real-world tasks require not only knowing what is where, but who is where: for example, a school teacher must generally know where different students are in a classroom and would use assigned seats to simplify such a representation. Social perspectives change spatial perspectives. Our language reveals how social relationships are mapped onto spatial ones: a *close* friend vs. a *distant* relation. These mappings occur on a societal level as well. For example, an American sample was queried about their social attitudes toward other nationalities and their spatial estimates of the distance of different cities in those nations. Participants with negative attitudes toward Mexicans overestimated how far away south from America the cities were; similarly, those with negative attitudes toward Canadians overestimated how far north Canadian cities are (Kerkman et al., 2004).

### Self movement perception and personality

Social perspectives can influence spatial cognition on large scales that are more abstract. Moreover, there are suggestions that social aspects of the self, influence even more basic aspects of spatial cognition at perceptual levels as well. Navigation for a sighted person appears to be so simple, that it can be done seemingly unconsciously. One key form of information used is optic flow, the changing angular movement of points, objects, and surfaces across the retina as one moves through the environment (Esch et al., 2001; Warren et al., 2001). The presentation of optic flow information on a screen when a person is not moving, as one might view moving stars in a film for instance, also elicits the perception of self-motion in humans; this is termed vection. Although much is known about how to produce vection in humans with such spatial, dynamic sensory information, less is known about how aspects of the self that might interact with the experience of self-motion as a form of embodied cognition, though there is tantalizing evidence that a relation exists. Seno found a relationship between vection and personality: narcissistic people were less affected by motion, presumably because they are more self-focused than environmentally aware (Seno et al., 2011). If one's self is the reference frame through which the world is perceived, then a more narcissistic perspective would give less weight to external stimulation, with a greater bias toward internally generated perceptual states. This provides an interesting avenue for future research to assess the application of models of interoception, the perception of the physiological condition of the body (Craig, 2002), to narcissism and perception. That is, this account suggests that narcissistic people are better in interoceptive tasks, and worse in exteroceptive tasks. This also suggests that people less sensitive to vection would have a stronger experience of interoception. In the same manner that narcissism can directly influence perception of the world, so might general states of enhanced perception of the self (Tsakiris et al., 2011). There is evidence of influence of brain areas that process emotion, empathy, and self-awareness on perception in general, and in psychopathology (Frith and Frith, 2003; Decety and Jackson, 2004; Seeley et al., 2007; Seth, 2013), suggesting this might be a fruitful line of further investigation.

## Social and spatial cognitive neuroscience

### Neurophysiology of self-mapping and navigation

**Figure 3:**
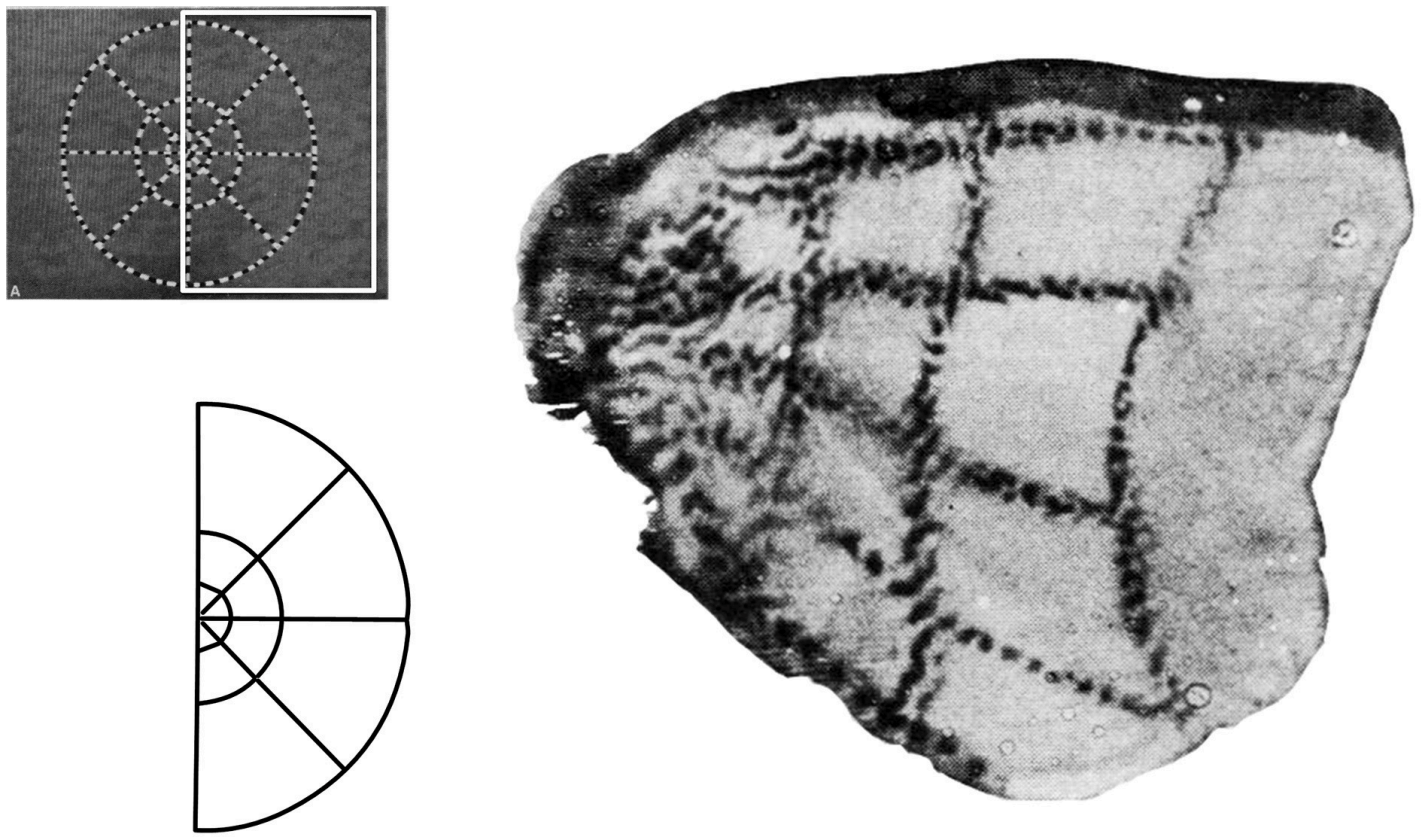
**Retinotopic maps are cortical representations of locations on the retina, with some distortions arising from the over-representation of the fovea compared to the periphery**. Here this is represented by a use of tracers injected in the retina that then demarcate regions of visual stimulation in the visual cortex. The visual stimulus is shown at the upper left; the white box shows the region of the stimulus that is represented in this area of the brain, with the lower left example showing this shape more clearly. The right image is of the stained cortex that represents this shape. Modified from Tootell et al. (1982).

**Figure 4:**
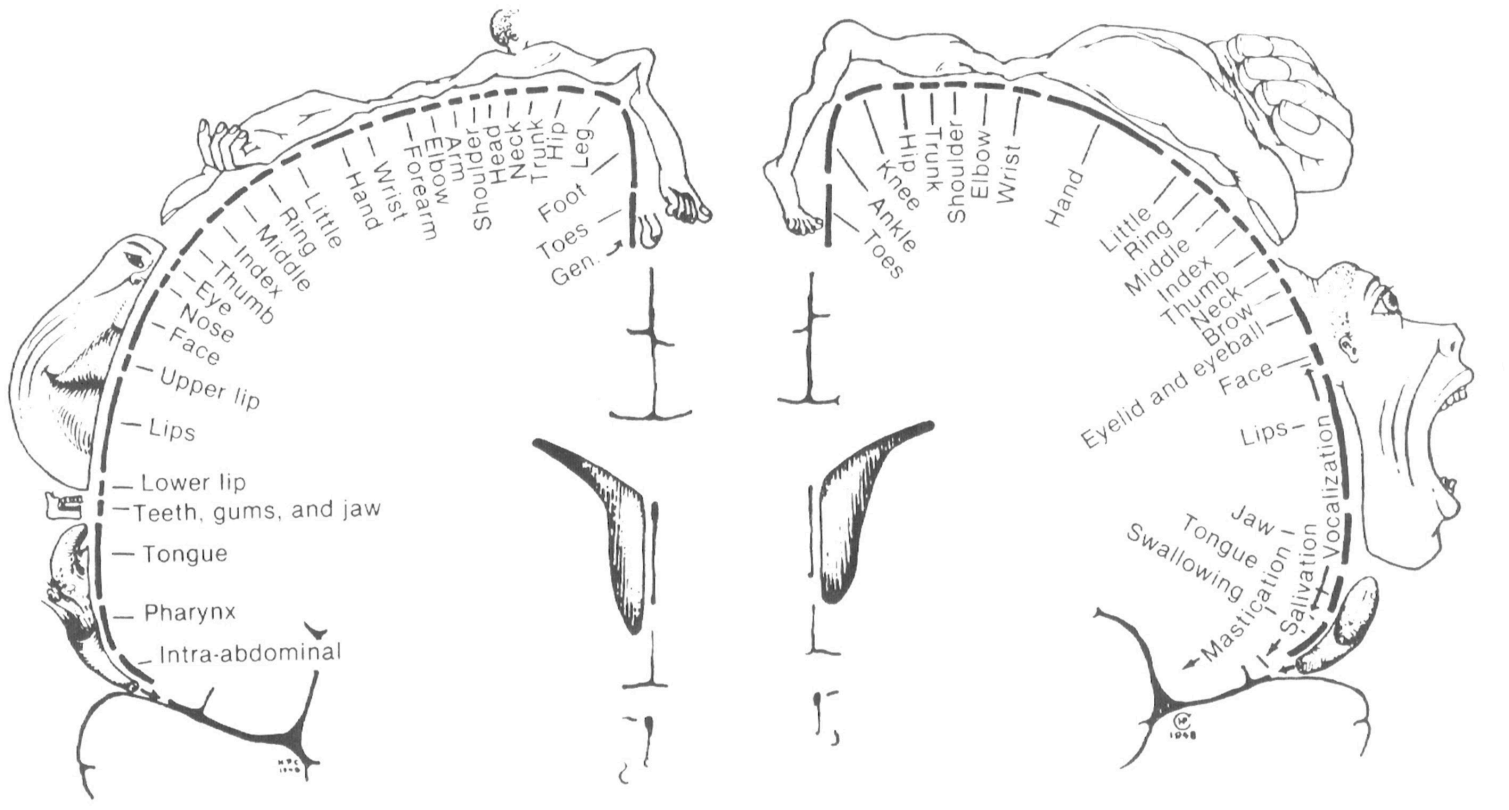
**The Penfield Homunculus: a visual representation of the mapping of body space in the somatosensory cortex of the brain, with the size of the body representing the size of the area of cortex devoted to it, and hence the sensitivity of that region as well**. From Penfield and Rasmussen (1950). THE CEREBRAL CORTEX OF MAN. © 1950 Gale, a part of Cengage Learning, Inc. Reproduced by permission: www.cengage.com/permissions.

Two major systems of spatial cognition are self-mapping and navigation, and each are crucial domains for understanding the role of egocentric and allocentric reference frames. In the *topographic maps* of sensory and motor systems, there is a direct correspondence between spatial coordinates in the body, and geometric coordinates in the brain. In each case spatial coordinates from a given sensory or motor surface (e.g., retina of the eye, skin on the body) are projected on to regions of the central nervous system. Within the central nervous system, the relative organization of the coordinates is maintained, and this organization is further re-projected in inputs to higher order brain regions. For example, the well-studied retinotopic maps originate from the retinal surface and occur in regions receiving visual inputs (Hubel and Wiesel, 1968). Spatial maps of the image on the retina are replicated first to pre-cortical regions (e.g., lateral geniculate nucleus, superior colliculus), with some inputs going on to the primary visual area which process visual information about the environment. In these regions, an orderly arrangement of visual inputs from the retina of the eye is clearly represented by the locations of the neurons (Figure 3). Retinotopic maps are also replicated in areas receiving inputs from V1, such as higher order visual areas and also multisensory areas of the PPC (Sereno et al., 2001). Typically, a single functionally-defined visual area is considered to be limited to one full retinotopic map (Sereno, 1995). Particularly relevant to the concepts of space and the self are the somatotopic maps of somatosensory and motor systems, which represent whole body coordinates (Penfield and Boldrey, 1937). The primary somatosensory cortex is a single cortical area representing the entire surface of the skin. A common pictorial representation of this is a cortical homunculus (see Figure 4). There, apparent distortion of anatomical regions in the image is because the location and sizes of individual anatomical regions reflects the innervation of those regions rather than overall size. This pattern of cortical magnification, in which anatomical regions which have greater sensation are better represented in the brain, can extend to other sensory modalities as well (Catania et al., 2011).

The region of space to which a given neuron responds to stimuli is called its *receptive field*. A receptive field is a property of neurons in both low level and multisensory CNS regions. In some higher order brain regions, inputs from multiple sensory modalities overlap. Thus, some neurons incorporate information from visual and tactile receptive fields, which are mapped onto body-centered coordinates. A multisensory neuron may respond independently to either a tactile stimulus within its tactile receptive field (e.g., air blown onto a spot on the face) or a visual stimulus within its visual receptive field (e.g., the appearance of an object looming toward the same spot on the face). The visual receptive field, like the tactile receptive field, is mapped onto body-centered coordinates, and thus maps visual space into body-centered reference frames (Graziano and Cooke, 2006). Somatotopic maps may represent not just the body itself, but also objects in the space nearby; they can be extended to include a tool (see below) and also can represent the body of another (see Section Interacting Neural Representations of Space and Self). *Personal space* is a similar notion which arose from social anthropology (Hall, 1963) and animal behavior (Hediger, 1950, 1955) and is usually defined as the space around the body which feels discomfort when it in entered into by another (Hayduk, 1978). *Peripersonal space* is space on and near the body the body for which there is corresponding activity of neurons that represent its coordinates (Rizzolatti et al., 1981). Peripersonal space is flexible in that when a tool is being used by an animal, the animals' receptive field, and peripersonal space, is enlarged to incorporate the tool (Maravita and Iriki, 2004). Personal and peripersonal space have been studied in social and non-social contexts and have been associated with distinct neural correlates (Teneggi et al., 2013). That is, regulation of personal space has been proposed to be a key role for the amygdala (Kennedy et al., 2009), whereas peripersonal space (specifically visual space around the face) is represented in the ventral premotor cortex [area 6 Brozzoli et al., 2011, 2012], putamen, and the PPC (Sereno and Huang, 2006), a system which seems to be more generally involved in defense and danger avoidance (Graziano and Cooke, 2006; Sambo and Iannetti, 2013), with some suggestions that different peripersonal representations might exist independently for approach and defense (Rizzolatti et al., 1997; Cléry et al., 2015; de Vignemont and Iannetti, 2015) Interestingly, peripersonal space seems to be built from the bottom up (cognitively impenetrable) as it is possible for a subject to be aware of a stimulus which should alter peripersonal space, and yet the peripersonal space is not altered (Hoogenraad et al., 1994; Kitagawa and Spence, 2005).

**Figure 5:**
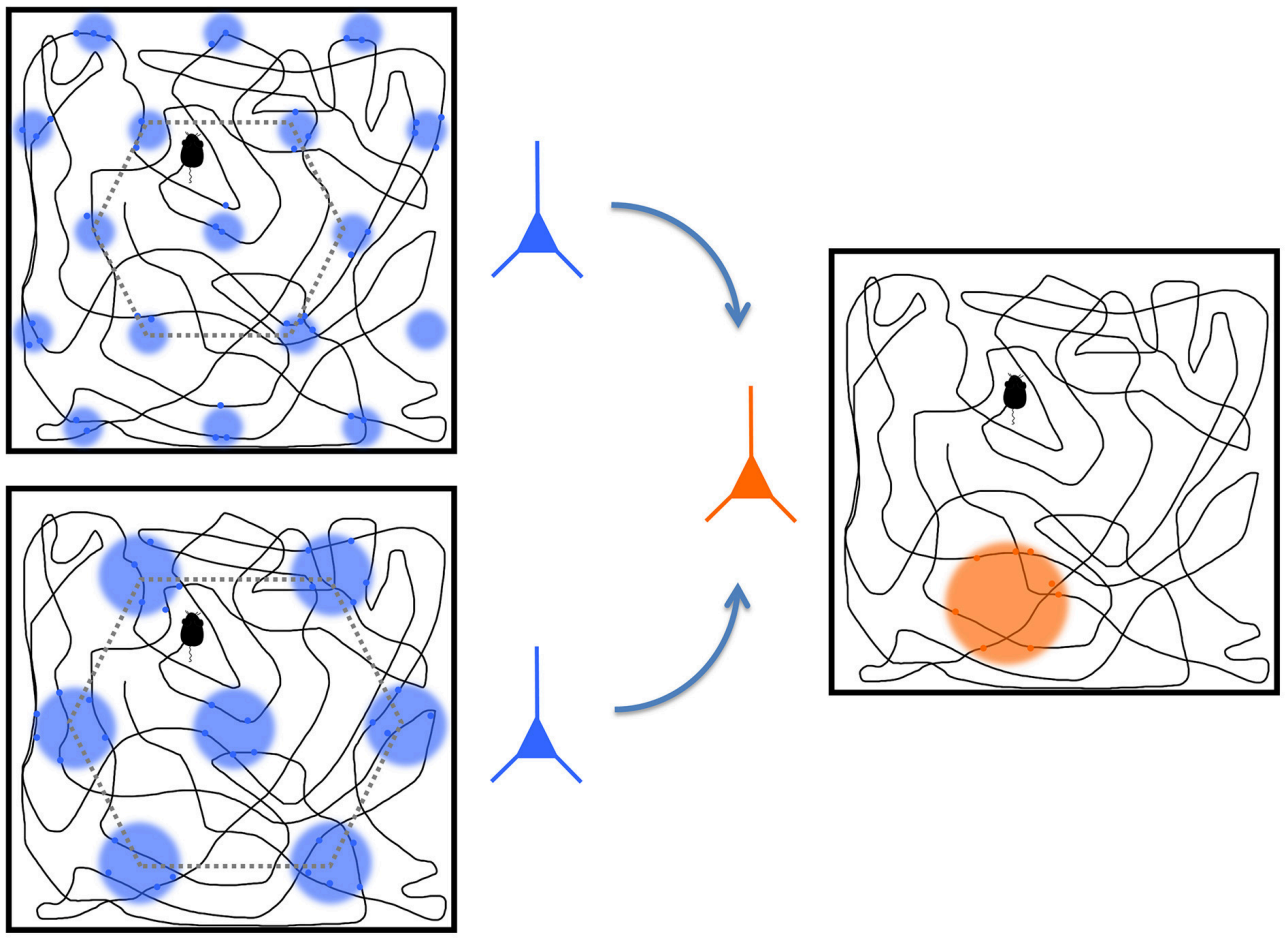
**A representation of two grid cells (blue) and a place cell (orange)**. The boxes show trajectories of a rat throughout a space with colored circles representing the regions where the cell fires the most. The grid cells, located in entorhinal cortex, operate at different spatial scales, and they project to the place cell in the hippocampus. The place cell convolves the responses from the grid cells to derive its preferred place at which it responds most strongly. Modified from Solstad et al. (2006) and from Nobelprize.org. (2014).

The neuroscience of spatial navigation is known largely from studies of freely moving rats. The *hippocampus system* has a declarative memory function that is also important to spatial perception and navigation in particular (O'Keefe, 1976; Moser et al., 2008). Whereas, the sensory and motor regions, with afferents coalescing in multisensory neocortical region such as the PPC, provide a frame of reference to the body' and its anatomical and sensory regions, the hippocampus maintains an updated representation of space and the animal's position within it. The hippocampus is a primitive seahorse-shaped limbic structure in the medial temporal lobe, is the neuroanatomical structure most often associated with spatial navigation due to the mechanisms that support remembering locations. It contains a population of pyramidal neurons, called “place cells,” that respond whenever an animal is in a specific location (see Figure 5; O'Keefe, 1976). Together these produce a dynamic cognitive map of the environment by firing in a concerted fashion (O'Keefe and Dostrovsky, 1971). The neighboring entorhinal cortex is responsible for the majority of cortical inputs to the hippocampus and provides the geometric basis of spatial perception in the hippocampus. An entorhinal “grid cell” is active when a freely moving animal enters a series of locations in an environment that the cell represents with a consistent geometric pattern (Hafting et al., 2005). The grid cells therefore provide a way for the brain to structure spatial information experienced through proprioception, which is necessarily serial in acquiring spatial information unlike retinotopic maps in visual cortex that receive spatial information in parallel. Unlike retinotopic maps, the organization of grid cells within the cortex is not well understood and does not reflect environmental topography (Hafting et al., 2005). Instead the connections between grid cells in the entorhinal cortex and place cells in the hippocampus gives rise to the representation of spatial maps that allow specific declarative memories to be associated with specific spatial locations represented by the place cells, which then might provide the basis for allocentric reference frame representation.

The hippocampal and topographic coding systems work together in spatial navigation tasks. It has been suggested that they link in parietal association areas of the neocortex, which are also crucial to spatial perception, contain topographic maps representing multiple sensory modalities, and may also provide navigational information (Arbib, 1997). However, the interplay between this neural network and that of the hippocampus are poorly understood (Whitlock et al., 2008). It has been suggested that parietal representations provide an egocentric frame of reference, and may map movements along a route according to route-centered positional information (Nitz, 2009). Similarly, in neuroscience, body-centered (or body-part-centered) space which is re-represented in the brain in an topographically organized manner is sometimes dubbed “egocentric space” (Schindler and Bartels, 2013), with the implication that this is the basis for an egocentric spatial perspective. In contrast, the role of the hippocampus in navigation, with its mechanisms of representing specific places in long term memory, has been hypothesized as the basis for allocentric frames of reference that are survey based rather than route-centered. The term “allocentric space” is sometimes used to describe a region of space as represented in an allocentric spatial task involving the hippocampus (Shrager et al., 2007).

### Interacting neural representations of space and self

The increasing psychological evidence for relations between social and spatial cognition are matched by new findings that suggest overlapping neural networks for processing these seemingly independent domains. Spatial cognition has many components, ranging from low-level retinotopic mapping to high-level spatial reasoning. General spatial awareness is relevant for social interactions, in that knowing who is where in relation to oneself is a core aspect of social behavior (Cheney et al., 1986). The parietal and temporal cortices are both involved in spatial awareness, as found in neuropsychological studies of neglect, neuroimaging research, and neurophysiological recordings (Halligan et al., 2003). The parietal lobe's role in various aspect of spatial processing, particularly for directing spatial attention, is well known (Culham and Kanwisher, 2001). Parietal cortex also plays a role in social interaction, yet with a particular spatial role. Neurophysiological recordings in macaques during social interaction found that the representation of space in parietal neurons was influenced by social interaction, particularly by the relation of the other's movements in relation to one's own movements (Fujii et al., 2008). In fact, the coding of space by parietal neurons was most influenced during times of conflict rather than just the mere social presence of another (Fujii et al., 2007).

In some neocortical areas, a neural representation of one's own body can map the body of another individual as well. Seeing another person being touch activates primary somatosensory cortex, overlapping with somatotopic organization for one's own body (Blakemore et al., 2005). In the mirror neurons of the macaque ventral premotor and inferior parietal cortex, an individual neuron can represent both one's own movement, and the same movement observed by another (Rizzolatti and Craighero, 2004). Because of its ability to map the other onto the self, the mirror system has been suggested to be the basis of empathy, with a central role in the evolution of the human social behaviors such as imitation and language (Arbib, 2005). Similarly, there is direct correspondence between spatial maps and social cognition in the ventral intraparietal area, a multisensory region with a head centered reference frame (Stepniewska et al., 2005; Sereno and Huang, 2006). A study in macaques found neurons which share a mapping of own visuotactile body coordinates and the body coordinates of others' bodies in a mirror-like fashion (Ishida et al., 2010).

The involvement of the temporal lobe and the temporoparietal junction in particular, provides another area of overlap between spatial and social processes (Karnath et al., 2001). As noted previously, the temporoparietal junction is implicated in spatial awareness (Halligan et al., 2003). The right temporoparietal junction has also been found to be crucial for numerous aspects of social interaction, from low-level attention to high-level understanding (Decety and Lamm, 2007). Some researchers argue that the right temporoparietal junction has a particular specialization for mentalizing, or the understanding of the mental states of another, in Theory of Mind tasks (Saxe and Powell, 2006). Although the relations between social and spatial processing have been examined in the parietal lobe (Fujii et al., 2008), the overlapping representations in the temporal lobe suggest that such interactions between space and self might also be present in the temporoparietal junction. In fact, work by Blanke and colleagues suggests the temporoparietal junction indeed plays a role in representing one's body in space. Some of his team's work used virtual reality to disrupt normal perception to create an out-of-body experience through the manipulation of multisensory cues (Blanke et al., 2002, 2005; Ionta et al., 2011; Blanke, 2012), thus impacting the spatial awareness of oneself.

### Neural correlates of personality and the social self

Numerous brain features have been put forth as possible correlates of personality, although the particular functions of these are often obscure. One study found an association between neuroticism and the ratio of whole-brain volume to intracranial volume (Knutson et al., 2001), opening up the possibility that some general anatomical properties might be behind certain personality differences. Another study found that self-described extraverts, when compared to self-described introverts, have thinner cortical gray matter in regions of the right inferior prefrontal cortex and fusiform gyrus (Wright et al., 2006)—areas related to recognition, risk aversion, behavioral inhibition and language comprehension. According to the same study, individuals who described themselves as more neurotic have a thinner cortical mantle in anterior regions of the left orbitofrontal cortex (Wright et al., 2006)—a region involved in decision-making and regulating expectation. It is not clear whether the neuroanatomical variables cause the differences in extraversion and neuroticism, or inversely, whether the personality traits result from alterations in regional brain anatomy, which are in turn caused by differences in lifetime experiences (such as social and affective experiences; Wright et al., 2006). Previous research has already pointed to the second possibility (Kolb et al., 2003; Als et al., 2004) and thus, further research into the topic might shed some light on the causal relationship between personality traits and anatomy. Another study found sex differences in the anatomical representations of extraversion and neuroticism (Blankstein et al., 2009). Blankstein and colleagues discovered that in females, extraversion correlated negatively with medial frontal gyrus gray matter volume, and neuroticism correlated positively with subgenual anterior cingulate cortex gray matter volume and cortical thickness (Blankstein et al., 2009)—regions often associated with social cognition and emotional processing. Surprisingly, the same traits were inversely correlated with the same brain regions in males, raising the possibility that sex differences in neuro-maturational divergence might be behind these relationships.

The search for the neuroanatomical correlates of the social self has proven to be extremely difficult, mainly because there are substantial disagreements as to what exactly is the self. As described above, following a more spatial, or embodied definition of the self, the physical self is mapped onto cortex in a spatially organized fashion, and the mapping of other's body can be superimposed over such a representation. Other neuroanatomical correlates have been suggested using other definitions of “self” centered on its narrative, conceptual properties. Upon defining the self as “temporally stable, trans-situational consistencies in behavior, dress, or political or religious ideology” one study in patients with frontotemporal dementia found that autobiographical memory and motivation have anatomic representations in the frontal lobes (Miller et al., 2001). In such patients, change in the self was observed as a shift from a previously well-defined self to a new well-defined self, and these changes could have been related to changes in the frontal lobes. This is supported by the finding that the maturation of self develops with myelination of frontal structures (Bower and Gilligan, 1979) and specifically the right hemisphere and the frontal lobe (Levine et al., 1998; Craik et al., 1999). An attempt to pin the self to brain structure more specifically has identified a region of medial prefrontal cortex that was selectively engaged during self-referential processing (Kelley et al., 2002).

## Evolutionary perspectives

### Evolutionary ecology models for the built environment (spatial and social cognition and individual differences)

Spatial and social behaviors are intertwined in many species, and the evolution and ecology of these behaviors provide many insights—indeed a foundation as illustrated in Figure 1—for their psychological and neural bases, and optimal application to the built environment. There are two main classes of explanatory factors regarding causation in evolution as postulated by Mayr—proximate and ultimate (Mayr, 1961). The former class deals with immediate factors acting upon the organism (i.e., physiology), while the latter is concerned with evolutionary factors working over generations (such as natural or sexual selection). Such distinction has served its purpose in facilitating thinking about various issues in biology, such as niche construction, cooperation, and the evolution of language (Laland et al., 2011; Scott-Phillips et al., 2011) among others, and is undoubtedly useful in thinking about the evolution, and development of various human specific behaviors. While psychology and physiology can answer multitude of questions related to the nature and development of spatial cognition, personality and the self, addressing, and answering the ultimate question about why these phenomena might be linked can be done solely through the scope of evolutionary theory. Further, evolutionary biology offers a broad framework which not only informs about our own species' past and future, but has the additional advantage of informing us about other organisms which make up the world we live in. A consideration of the effect of the environment on the evolution of behavior, anatomy and physiology—in general and in the emergence of modern *Homo sapiens* in particular—is crucial for prescribing architecture for human wellbeing.

The notion that the human-built environment can influence the course of evolution in *any* species has been gaining support in recent studies (Hunter, 2007) and has implications about the importance of the built environment for humans in particular. Several species may be adapted to urban environments: city spiders are larger, urban rivers salmon are smaller, city earthworms are more tolerant to metals, urban plants disperse fewer seeds (Alberti, 2015). Urban songbirds have acoustic signals and personalities better suited to the urban environment: European blackbirds in particular switch to more sedentary lifestyle and modify their migratory behavior in response to urbanization (Partecke et al., 2006; Miranda et al., 2013). If such human-mediated ecosystem changes lead to rapid changes in the course of evolution of other species, then recent changes have likely been occurring in our species, too. Understanding the evolutionary pressures acting upon human and other species' behavior and anatomy is critical in order to make urban ecosystems favorable to our wellbeing. Thus, in this following section we outline the contribution of both spatial and social pressures in shaping primate evolution in an attempt to shed light on some of the evolutionary pressures that led to the evolution of modern humans.

Advancements in both spatial cognition (Parker and Gibson, 1979; Baumeister, 1987) and social cognition (Jolly, 1966; Humphrey, 1976; Wynn, 1985) have been implicated as important factors in shaping a multitude of human behaviors. However, these are often posed in opposition to each other as drivers of cognitive evolution (Boyd and Silk, 2009). Decades ago, Milton (1981, 1988) emphasized the importance of spatial cognition in primate and human evolution—a notion called the “ecological intelligence” hypothesis. It suggests that different ecological challenges (such as extractive foraging, diet breadth, seasonality) are main drivers of cognitive evolution in that animal order. However, this idea has fallen into disfavor in recent years, due largely to the popularity of the “social intelligence” hypothesis. In the “social intelligence” hypothesis, Dunbar et al. (Dunbar, 1992; Dunbar and Shultz, 2007) suggested that sociality (indicated by group size) drives cognitive evolution and advanced intelligence, and is also a determinant of relative neocortex size. The debate is far from settled, for example the social intelligence hypothesis was recently challenged by MacLean et al. (2014), who suggested that social group size was unrelated to brain size and intelligence (represented by the executive function of self-control) and pointed to foraging challenges (diet breadth) as a better predictor for cognitive performance within the primate order (MacLean et al., 2014).

In fact, the view we support here is that social and spatial factors were both prime movers in primate cognitive evolution, and likely co-evolved inter-dependently for behavioral and neuroanatomical reasons. First, primate foraging posits cognitive demands beyond those of other animals, and can include long-distance movement through a three dimensional arboreal environment, the acquisition of rare and high-quality foods, tool use, the carrying of young, and communication (Janmaat et al., 2013; Ban et al., 2014). The evidence so far suggests that primates owe their large brains and advanced cognition to finding food (foraging challenges), and that neural and cognitive features were then later co-opted for social behavior (Zuberbühler and Janmaat, 2010).

Second, the neuroanatomical substrates for spatial cognition may have been co-opted for social cognition, perhaps very early in the mammalian lineage, and thus the trend would have already existed well before the primate radiation. Brain areas which represent space topographically are conserved across vertebrates, and more recent cortical areas with similar organization have been proposed as the basis of the most complex primate social behaviors. For example, retinotopy is a feature of the tectum (superior colliculus) which is a primitive structure in the primate brain that uses retinotopic maps to guide eye saccades and other movements body parts in space (Lane et al., 1973). The retinotopic organization is evolutionary conserved and is also seen in the most primitive non-mammalian vertebrates in which the optic tectum is the primary structure directing visually-guided movements (Cornide-Petronio et al., 2011; Gandhi and Katnani, 2011). Similarly motor neurons tend to be arranged somatotopically in vertebrate brains [Graziano and Aflalo, 2007; even though this is not always necessary for complex movements Zullo et al., 2009]. The somatotopic organization of body parts in the cortex could allow for their coordination in complex movements (Graziano et al., 2002). Topographic organization in sensory and motor modalities is also maintained in the mirror neuron system in which neurons are active both in conducting a movement of oneself, and in seeing the movement demonstrated by another (Rizzolatti and Craighero, 2004). There is some debate surrounding the specific reference frames that mirror neurons employ, such as an abstract representation of space independent of modality (Rizzolatti and Arbib, 1998), a somatotopic organization of responses that mirror what is seen (Buccino et al., 2001), or changes in processing as a function of whether the actions are within or outside the animal's peripersonal space (Caggiano et al., 2009). Regardless the role of spatial topography in complex movement would extend to all of these mirror system behaviors, even if the specific topography differs. The mirror system could have set the basis for behaviors of empathy and imitation (Iacoboni, 2009). It is suggested to have evolved in function in seven stages coinciding with increasing social complexity, starting from “cortical control of hand movements (S1),” “A simple imitation system for grasping shared with common ancestor of human and chimpanzee” (S3), and eventually language (S7) (Arbib, 2007). As mentioned above, neocorticalization is key trend coincident with increasing social complexity across species; neocortical areas could also expand to represent space with higher acuity (de Sousa and Proulx, 2014), or in more modality-specific domains (Changizi, 2001).

There may also be overlapping trends in the evolution of spatial and social cognition in the hippocampal system, which is involved in both. The ecological variable best associated with spatial cognition and used to compare behaviors across species is the size of the home range, defined by Burt as “that area traversed by the individual in its normal activities of food gathering, mating, and caring for young”(Burt, 1943). It has been suggested that home range is a driving factor of mental ability generally (Milton, 1981), although more particularly it is thought that home range determines spatial navigational demands, and spatial navigational demands drive spatial ability (Jacobs et al., 1990). In fact, Powell and Mitchell (2012) define home range cognitively, in that “home range is that part of an animal's cognitive map of its environment that it chooses to keep updated.” Studies have suggested a direct link across species of rodents (Jacobs and Spencer, 1994), and birds [the “avian hippocampus” is the medial pallial zone Rodríguez et al., 2002; Krebs et al., 1989; Sherry et al., 1989; Garamszegi and Eens, 2004; Lucas et al., 2004] between advanced spatial behaviors, including maintaining large home ranges and remembering and find food caches, and the average size of the hippocampus. Also in humans, there is interindividual variability in ranging, which is linked to hippocampal function: the volumetric reorganization of the hippocampus has been directly related to the occupational specialization in spatial memory, as with the increased size of the hippocampi of taxi-cab drivers (Maguire et al., 2000). The hippocampus plays a critical role in memory consolidation (from short term to long term memory; Squire, 1992; Squire and Alvarez, 1995), and has minor roles in behavioral inhibition (Taylor et al., 2014) and olfactory memory (Kaut et al., 2003). In addition the hippocampus may be split into distinct social and spatial parts. Recently, CA2, an obscure subregion of the hippocampus for which little was functionally known, has been implicated in the encoding of social memory (Hitti and Siegelbaum, 2014), and for attacking male intruders (Pagani et al., 2015), although it does not code for space in the same way as other hippocampal subregions (Mankin et al., 2015). It is also the case that in a task in which humans have to bring up knowledge of social networks the anterior hippocampus was selectively activated (Kumaran et al., 2012), although the same neural networks are not known to be involved in navigating social and spatial networks (Kumaran and Maguire, 2005). Interestingly, these novel findings provide direct evidence for the interrelatedness of both the social and spatial aspects of memory. Dunbar speculated that the number of neocortical neurons is a limiting factor in determining the number of social relationships which an animal can monitor (Dunbar, 1992); given the role of the hippocampus in social memory in particular it should also be an important consideration.

Additionally, individual differences have been shown to be present in numerous vertebrate and invertebrate species and to have fitness consequences (Réale et al., 2007). While genetically-encoded conditional strategies are useful adaptations in the face of highly predictable changes (Stephens, 1991), individual differences, and different experiences, can serve to shape the organism's response to unpredictable or fast change (Shettleworth, 2010). For example, individual differences in search strategies have been shown to be consistent within many species (Giraldeau and Dubois, 2008) and in some they are part of broader behavioral syndromes including aggressiveness, the propensity to use social cues, and learning performance (Marchetti and Drent, 2000). In some species it has been shown that individual differences underlie the tendency to use own experience vs. learning from others (Giraldeau and Dubois, 2008). In that respect, the evolution and maintenance of personality differences plays a major role in determining individuals' proclivity to use social information, and has important fitness consequences (Reader, 2015). Another example points to the importance of early life experience in shaping individual differences in spatial cognition: in egg-laying hens (*Gallus gallus domesticus*), individuals raised in cages performed slower and showed impaired working memory in comparison to aviary raised chicks in hole-board exploration task (Tahamtani et al., 2015). Similarly, the role of experience in shaping the preferred spatial reference frame in humans has been shown to depend largely on cultural background (Goeke et al., 2015). These findings suggest that genetically encoded conditional strategies serve their function in maintenance of individual differences, but personal experience is a powerful proximate cause for such differences. When faced with a rapidly changing environment, such as a human-built environment, individual differences can influence both spatial and social cognition, and this can have fitness consequences.

### Spatial and social cognition in human evolution

Advancements in tool making and brain reorganization consistent with improved spatial cognition appear early in human evolution, and continue through to the emergence of our species (de Sousa and Cunha, 2012). As indicted above, primates have high demands for spatial ability. Additional demands on human spatial ability include tool making, large home ranges, and migration. These early behaviors may have depended on novel social communication attributes such as empathy and language. For example, the ability of humans to manufacture tools has been closely linked to our ability to communicate socially with one another (Stout and Chaminade, 2009). An experimental study found that teaching and language were likely involved in the production of Acheulean tools, the first tools exhibiting spatial conceptualization due to symmetry and form consistency (the technology persisted across much of the Old World and covered a time period of ~1 million years; Morgan et al., 2015). Migration and large home ranges may put individuals at increased risk for predation and environmental stressors. In non-human primates these stressors may be accommodated for by an increase in social group size (Hill and Lee, 1998), but migration and home range expansion literally move conspecifics away from each other. Therefore, major advancements in social communication such as language and teaching could be even more important when fewer social relations are available. Also, major advancements in spatial cognition might have been vital to colonizing new ecological zones. The earliest human shelters, constructed during the Paleolithic (de Lumley, 1966), would have been instances in which building environments would have enabled hominins to define and recreate spaces in three dimensions for novel purposes.

Tool-use and tool-making place special demands on spatial cognition at the level of the individual, as well. When an individual uses a tool it becomes an extension of the self as it is incorporated into pre-existing topographic map of the body, something well studied in higher primates (Iriki et al., 1996). Additionally, humans makes tools which do not directly resemble the substrate from which they are made, thus tool making has been suggested to place demands on spatial cognition not found in other species (Wynn, 1985). Functional neuroanatomical studies in humans have revealed shared neural systems for tool production and language (Stout and Chaminade, 2009; Uomini and Meyer, 2013). The motor acts of speech and tool-making are both hierarchically structured sequences of behavioral “units”; thus it has often been wondered whether there is any meaningful correspondence between these (Holloway, 1969). It has been posited that in the human lineage neural systems originally serving in visuotactile-spatial function were later co-opted for a particularly social function: human language. The mirror system hypothesis draws upon the putative homologous relationship between the monkey area F5, which is a hand motor area containing mirror neurons and involved in tool use, and the human Broca's area, involved in motor control of oro-laryngeal movements and speech production. According to this model, the mirror system for matching observation and execution of hand movements sets the basis for social communication and eventually human speech (Rizzolatti and Arbib, 1998).

Early transitional hominins have been suggested to exhibit a shift to upright, bipedal posture (Lovejoy et al., 2009). Although the timing and nature of this transition is hotly debated, what is clear is that hominins became habitually bipedal and colonized a vast geographic range well before they demonstrated fully modern behavior, cognition, or brain size (de Sousa and Wood, 2007; de Sousa and Cunha, 2012). The earliest dated evidence of hominin intercontinental migration, for example, comes from *Homo erectus* at the surprisingly early site Dmanisi, in Georgia, 1.8 million years ago (Finlayson, 2005). It is only possible to speculate about whether spatial navigation strategies may have emerged to enable early migrations. However, compared to our closest living ancestor, the chimpanzee, humans possess a tool which can greatly facilitate spatial navigation: the physical map. Maps are “the symbolic system par excellence for encoding and permanently retaining spatial information” (Landau and Lakusta, 2009). Creating a map requires making spatial transformations, which are symbolic and yet they are analog to what they represent, unlike language (Landau and Lakusta, 2009). It has been suggested that the earliest map was created and used by members of our species: an engraved piece of ivory at the Upper Paleolithic site Mezhirich in the Ukraine (Bahn and Vertut, 1997). Furthermore, the capacity to use a map-like object, such as a scale model, might be a human-specific ability; though only one study has attempted to assess this, and failed, in chimpanzees with a complex task that might have required additional abilities (Kuhlmeier and Boysen, 2001). Although the data are few, attempts have been made to interpret prehistoric hominin space use patterns (Wren et al., 2014) in order to understand human specific aspects of spatial cognition, and in particular to seek evidence of egocentric and allocentric strategies (Burke, 2012). It would be a hard task to study individual differences in human fossils, and how these may relate to spatial cognition. Yet, it is possible to compare different groups of fossils, such as species or populations. Like modern humans, our close fossil relatives the Neanderthals structured domestic spaces into distinct zones (Riel-Salvatore et al., 2013). Still, it is suggested that modern humans might have had advantages in spatial navigation, even compared to the Neanderthals, and that sexual selection has played an important role in the evolution of spatial cognition (Burke, 2012).

## The built environment

### Environmental influences on spatial reference frames

How might design influence spatial cognition directly, and (perhaps) social cognition indirectly? Given the foundation in evolutionary theory, and the relation between the psychological concepts of space and self (see Figure 1), there are a number of personal and environmental variables that influence the reliance on egocentric and allocentric frames of reference (Ekstrom et al., 2014). First consider a number of environmental variables: (a) the size of the space; (b) the geometric structure of the environment; (c) the complexity of the environment; and (d) the landmarks available in the environment. Also consider a number of variables that appear to be more personal in terms of individual differences, though each of these can be influenced by the structure of the environment presented to an individual: (a) the manner of acquiring spatial information; (b) familiarity with an environment; (c) the duration of time spent in the space; (d) how one moves through a space; (e) the presence of social beings vs. objects; and (f) the personality of the individual.

The size of the space can clearly influence the reference frame used (Presson et al., 1989; Roskos-Ewoldsen et al., 1998). For example, if the space is smaller, and the organism has not had extensive experience with the space, then an allocentric representation might not be required, with even the hippocampal processing being unnecessary for small enough spaces (Day et al., 1999). Similarly, if one can see the area in question, having a “vista” of the locations allows one to rely solely on an egocentric rather than an allocentric reference frame (Meilinger et al., 2014). The geometric structure of the environment also influences the reference frame used. In work reviewed elsewhere here, the intrinsic structure of locations, say in ordered rows and columns, can determine whether egocentric or allocentric representations are used independent of the viewpoint one has had (McNamara et al., 2003). Other environmental influences include complexity and the existence of landmarks. If the organization of locations is more complex, then there might be a shift from egocentric to allocentric frames. Similarly, if there are clear landmarks, then an allocentric frame is possible; if there are none, then an egocentric reference frame dominates (Shelton and McNamara, 2001).

The environment can also be structured to influence a number of aspects of individual learning and experience that affect the spatial reference frames used. The way of learning spatial information impacts the reference frame used (Presson and Hazelrigg, 1984). For example, learning a new environment with a map leads to an allocentric representation, yet route learning is more likely to lead to an egocentric representation. One's familiarity with a space can influence reference frames, too. The greater familiarity one has with a place increases the knowledge one has of different perspectives and orientations, which affords an allocentric representation (Evans and Pezdek, 1980; Thorndyke and Hayes-Roth, 1982; Ruggiero et al., 2009; Iachini et al., 2014). Similarly, the amount of time one is exposed to an environment leads to a shift from an egocentric reference frame arising from momentary experience, to an allocentric reference frame based on additional experience. This also suggests that having unrestricted movement in the space over time allows for the experience of multiple paths and perspectives as well for gaining allocentric knowledge. As noted in prior sections, the presence of others, rather than just objects (Shelton et al., 2012), and the personality of the individual in the environment (Seno et al., 2011) are other individual differences that interact with the structure of the environment.

### Confinement in the built environment: Impacts on social and spatial cognition

We have thus far suggested how spatial factors interact with personality, individual differences, the social self, and perception. How might impositions on space from the built environment affect social and spatial cognition?

Incarceration is an extreme and long term restriction of movement in space which has recently received much political, social, and media attention. Whether in the sphere of prison reforms and attitudes surrounding punishment, or through the questions of how best to care for those with severe health problems, the questions surrounding the practice of “putting away” people deemed to be reprehensible, risky, or in danger is one which evokes strong feelings and generates much controversy and discussion.

Within philosophy, growing attention is being given to what might be interpreted as disorders of confinement such as claustrophobia (Trigg, 2012) and agoraphobia (Jacobson, 2004, 2011). In both instances, the person suffers from atypical constructions of confinement, felt as a constriction in lived spatiality. Recent work has explored how this structures the way a person engages with their environment, with other people, and with their projects (Heidegger, 1962; Merleau-Ponty, 1962; Husserl, 1989; Aiken, 2011; Krueger, 2013). Others have explored the way in which spatial construction influences and stems from “socio-political relations” (Foucault, 1973, 1976; Harvey, 1973; Lefebvre, 1991; Massey, 2005). However, there has not yet been much attention devoted to the effect which an *imposed* constriction of spatiality has on the individual, their personality and mental health, and also the way in which experience comes to be restructured for them. Likewise, though there has been much consideration of the ways in which our “situatedness” in a social world and embodiment structures both social cognition (De Jaegher and Di Paolo, 2007; De Jaegher, 2009) and intersubjective understanding (Gallagher et al., 2002; Gallagher and Brøsted Sørensen, 2006; Gallagher, 2007, 2008, 2015), there has been surprisingly little attention devoted to developing an account of the role which spatiality plays in both these spheres.

Human beings are always *spatial* beings (Heidegger, 1962), with a basic relationality in which spaces are shaped in relation to activities we are engaged and involved in. In Heidegger's treatment and exploration of “regions” of space in *Being and Time*, he writes that we are Being-*in*-the-world, in which the “in” designates a basic relationality in which spaces are shaped in relation to activities we are engaged and involved in Heidegger (1962) and Husserl (1989). Groupings of equipment become salient in the field of action, to create regions of activity. In this way, space is made significant by a person's projects. Merleau-Ponty's analysis of spatiality complements and builds on that of Heidegger to offer consideration of how embodiment shapes an experience of space. Merleau-Ponty argues that experience of spatiality is constructed in relation to our bodily abilities and limitations; our body throws open a world of possibilities which impact on the way in which we apprehend, move through, act within, and relate to our spatiality (Merleau-Ponty, 1962). Contrariwise, the question of how bodily limitations—through illness or incarceration—double back to effect the ways in which a person experiences the possibilities which are available and open to them must, then, be asked.

How would the restricted environment of an incarcerated person influence their perception for self? Long term inmates frequently report changes to their sense of *lived time* in which references to deep boredom, and feelings of damnation and despair abound. There is also a growing field of work which seeks to understand changes to temporality in varying forms of illness (Minkowski, 1970; Fuchs, 2012; Ratcliffe, 2012). It is important to note that incarceration is an example of involuntary confinement in a restricted space, often alone. What about voluntary confinement?

Living in space and space travel, such as planned missions to Mars, require long periods of confinement, though voluntarily accepted and often not in social isolation. A number of studies have examined how such confinement during actual missions influences one's perceptual and cognitive abilities. For example, distance estimation and size perception, such as that studied with visual illusions, are both impacted by the combination of a restricted environment and microgravity (Clément et al., 2012, 2013). Other studies have examined voluntary confinement through studies of submariners, who also do not have the windows that provide far-distance cues that astronauts have. Although the vision of submariners remained in the normal range, there was evidence for negative outcomes due to the restricted space such as increased myopia (short-sightedness), decreased accuracy in distance estimation, and muscular control issues (Kinney et al., 1979). Perception and memory for visual scenes have also been found to be impacted by time spent in a confined environment. Confinement to a space seems to impact the boundary extension effect. Normally, people exhibit boundary extension when remembering visual images; that is, people are more likely to recognize a wide-angle, zoomed-out, image as the one they saw rather than the actual image seen (Intraub and Richardson, 1989). Volunteers who spent 105 days in a windowless space, to study confinement of the sort expected with space travel, were tested on their ability to recognize images of scenes (Lukavský, 2014). Although control participants who were not confined exhibited no changes in the boundary extension effect, the effect increased in volunteers who were confined, likely due to the impact of experiencing such a small space for such a long period of time. Distance estimation is poorer when made in a confined space, like a corridor (Lappin et al., 2006), and it appears that long-term confinement influences perceived distance in two-dimensional images as well. Longer-term confinement might have even broader psychological implications. Similar studies of changes in visual cognition due to voluntary confinement of 500 days for a similar simulated space mission were difficult to study due to increased apathy and fatigue (Lukavský, 2014; Sikl and Simecek, 2014). The social isolation that accompanies incarceration might give rise to even greater difficulties.

The benefits of building designs can be compared in terms of quantitative data about health, stress, and other aspects of physiology (Edelstein, 2013). Hospitals provide an interesting format for studying this because patient health is monitored and patient mobility is limited. An impactful study found that post-surgical recovery time may be reduced with a window view (Ulrich, 1984). This finding is typically likened to Ulrich's “nature restoration hypothesis” based on a preference for natural scenes over urban ones, but could also have to do with the allusion to overall spatiality offered by a natural scene—and might help explain a particular preference for seeing water (Ulrich, 1979). In a study using virtual spaces, it was found that physiological stress responses are reduced in rooms which offer escape routes (Fich et al., 2014). Speaking of recovery of patients, Toombs writes “Whereas the restoration of wholeness may be limited in terms of restoring bodily integrity or eradicating ‘disease’, the physician can assist the patient in regaining control (even if it is only limited control), overcoming helplessness and thus retaining the freedom to act” (Toombs, 1992). These three areas of “control,” “overcoming helplessness,” and maintaining “freedom to act” are areas in which spatial planning and design can perform a key role. In characterizing the relationship between person and world as one which is necessarily shaped by, and through, bodily phenomenology, Merleau-Ponty presents the world in terms of the “I can.” Our bodies, in constraining the things that we experience as possible, impossible, challenging, comforting, and so on, opens up and presents possibilities for action and activity (Merleau-Ponty, 1962). For Merleau-Ponty this also affects our intersubjective relationships with others. Intersubjectivity is shaped through intercorporeality (Merleau-Ponty, 1962, 1964). In illness bodily transparency—which captures the way in which we do not ordinarily notice our body, which Merleau-Ponty argues is the norm of the “I can,” gives way to opacity and reconfigures experience in terms of “I cannot.” The body, instead of opening up the world, becomes an obstacle: things must be done in spite of it (Fuchs, 2005). This has potential to directly influence the way in which spaces are perceived, in particular as ones in which we may participate or not. The question then becomes how recovery spaces can be designed to lessen the magnitude of the “I cannot” in order to provide a feeling of “control” and ability to navigate the space, and daily tasks, successfully.

Urban planning and building design can reflect both the needs and culture of those using it, and also potentially affect the cultural and social experience of the inhabitants. Studies of confinement could give insight into how the perception of space is influenced by and influences aspects of the self-defined individual or as part of the culture the individuals come from. The research should also indicate how the non-visual perception of space and self-motion can influence the perception of space and magnitude, and thus be applied to buildings (real or virtual) created for the sensory or physically impaired. The layout and construction of a building might better promote successful navigation for those with sensory impairments (Passini and Proulx, 1988), and those inclusive design principles in turn might be better for everyone independent of having impairments or not (Passini, 1996).

### Spatial behavior: From architecture to neuroscience, and vice versa

Who we are is where we are. Our minds map ours bodies and the world around us. As discussed above, egocentric and allocentric reference frames are often studied as two opposing spatial strategies in navigation and other aspects of spatial cognition, however, these may actually be linked across social and spatial cognition. Individual differences in cognitive and sensory abilities impact navigation as well (Passini and Proulx, 1988; Marquardt and Schmieg, 2009; Marquardt, 2011; Pasqualotto and Proulx, 2012; Pasqualotto et al., 2013). Not only do aspects of the individual, or the self, impact spatial cognition, but properties of the social and physical environment can impact that as well. Certain built structures could invite different strategies in navigation, and may result in different perspectives of the self in relation to other objects. For example, we invite a reconsideration of confinement, in which the lack of mobility in space could also impair perspectives of the social and emotional self. A well-built environment can promote the best in well-being for positive health outcomes and social behavior (Aries et al., 2010; Lawson, 2010). Aspects of spatial cognition may be trademarks of the human experience that should be afforded to all, thus we advise built environments to address all abilities through inclusive design. Recently, architects and urban planners have begun to incorporate considerations of the abilities and references frames of those using the space to optimize design of the built environment to support successful navigation with inclusive design that considers how building for the visually (or otherwise) impaired might be best for everyone (Passini, 1996).

Our review has further implications for designing public housing spaces, social spaces, and even nurseries. The fact that experience can shape individual differences, which in turn can affect the quality of spatial and social cognition, suggests that growing up in certain built environments can have detrimental or beneficial effects on cognitive ability. For example, blocks of flats, which introduce a major third vertical dimension to navigation (Jeffery et al., 2013; Pasqualotto and Proulx, 2013), and at the same time providing limited spatial perception due to walls and other visual constraints, might play a major role in shaping cognition via both proximate and ultimate mechanisms. No study, according to our knowledge, has ever addressed the possible personality, spatial reference frame proclivity, or social cognition differences between block vs. house vs. open space dwellers. Additionally, nurseries, an early life environment which also has the ability to shape personality differences, and thus cognition, has never come into scrutiny regarding its architectural properties. Raising children in enclosed spaces vs. open spaces, or such with abundance of windows or glass walls, is supposed to result in differences in spatial and social cognition and is another issue worth addressing in light of this review. In terms of planning social spaces around public housing and elsewhere, an important point is again keeping in mind the interplay between spatial and social cognition and individual differences. Equal access to such places should be provided to everyone, disregarding bodily constraints. Planning for such accessibility would assure equal chances for “experience” for both able and disabled people, and provide equal opportunities for personal development. Another area which might benefit from further research in this direction is planning of spaces where political decisions are usually made—i.e., town halls or parliaments. The extent to which these provide constraints or enable individuals in spatial and social perception, in interaction with individual differences, might result in more effective policy making.

This is a solid start, but could be extended with further consideration of the interactions of spatial cognition and ability, personality, and differences in cognition. Where we are might mold who we are, but given our ability to shape the environment, we can play an active role in the development of the self.

## Funding

This work was supported in part by a grant from the EPSRC to MP (EP/J017205/1).

### Conflict of interest statement

The authors declare that the research was conducted in the absence of any commercial or financial relationships that could be construed as a potential conflict of interest. The reviewer AS and handling Editor declared a current collaboration and the handling Editor states that the process nevertheless met the standards of a fair and objective review.
