# Corrigendum: Where am I? Who am I? The Relation Between Spatial Cognition, Social Cognition, and Individual Differences in the Built Environment

**DOI:** 10.3389/fpsyg.2016.00554

**Published:** 2016-05-19

**Authors:** Michael J. Proulx, Orlin S. Todorov, Amanda Taylor Aiken, Alexandra A. de Sousa

**Affiliations:** ^1^Crossmodal Cognition Laboratory, Department of Psychology, University of BathBath, UK; ^2^European Network for Brain Evolution ResearchThe Hague, Netherlands; ^3^Department of Philosophy, University of DurhamDurham, UK; ^4^School of Society, Enterprise and Environment, Bath Spa UniversityBath, UK

**Keywords:** spatial cognition, social cognition, navigation, personality, reference frames, allocentric frame of reference, egocentric frame of reference, cognitive neuroscience

Reason for Corrigendum:

There was a mistake in the figure heading for Figure 4. The heading should note that the left panel is somatosensory and the right is motor cortex. The correct figure heading appears below. The authors apologize for the mistake. This error does not change the scientific conclusions of the article in any way.

**Figure 4. The Penfield Homunculus: a visual representation of the mapping of body space in the somatosensory (left panel) and motor (right panel) cortices of the brain, with the size of the body representing the size of the area of cortex devoted to it, and hence the sensitivity of that region as well**. From Penfield and Rasmussen (1950). THE CEREBRAL CORTEX OF MAN. ©1950 Gale, a part of Cengage Learning, Inc. Reproduced by permission: www.cengage.com/permissions.

## Author contributions

All authors listed, have made substantial, direct, and intellectual contribution to the work, and approved it for publication.

### Conflict of interest statement

The authors declare that the research was conducted in the absence of any commercial or financial relationships that could be construed as a potential conflict of interest.

